# Biodegradation of *p*-nitrophenol by engineered strain

**DOI:** 10.1186/s13568-021-01284-8

**Published:** 2021-08-31

**Authors:** Jing Xu, Bo Wang, Wen-hui Zhang, Fu-Jian Zhang, Yong-dong Deng, Yu Wang, Jian-Jie Gao, Yong-Sheng Tian, Ri-He Peng, Quan-Hong Yao

**Affiliations:** Shanghai Key Laboratory of Agricultural Genetics and Breeding, Agro-Biotechnology Research Institute, Shanghai Academy of Agricultural Sciences, Shanghai, 201106 China

**Keywords:** *p*-Nitrophenol, Degradation, Multigene metabolic engineering, *E. coli*, Bioremediation

## Abstract

*p*-Nitrophenol (PNP) is an important environmental pollutant and can causes significant environmental and health risks. Compared with the traditional methods, biodegradation is a useful one to completely remove the harmful pollutants from the environment. Here, an engineered strain was first constructed by introducing PNP biodegradation pathway via the hydroquinone (HQ) pathway into *Escherichia coli*. In the engineered strain BL-PNP, PNP was completely degraded to β-ketoadipate and subsequently enter the metabolites of multiple anabolic pathways. The high tolerance and rapid degradation ability to PNP enable the engineered strain to have the potential to degrade toxic substances. The engineered strain created in this study can be used as a functional strain for bioremediation of PNP and potential toxic intermediates, and the method of assembling aromatic hydrocarbons metabolic pathway can be used to eradicate nitroaromatic pollutants in the environment.

**Supplementary Information:**

The online version contains supplementary material available at 10.1186/s13568-021-01284-8.

## Key points

1. Five genes of PNP biodegradation pathway were synthesized and modified.
2. All genes were regulated by monocistronic transcriptional pattern.
3. PNP and subsequent decomposition intermediates have been completely degraded.

## Introduction

Nitroaromatic compounds have been widely used as dyes, pesticides, herbicides, plasticizers and explosives (Zhang et al. 2018). The presence of these nitroaromatic compounds and their subsequent release has led to severe environmental pollution of soil, ground water and air. Therefore, nitroaromatic compounds has been rated as priority pollutant (as HR-3 grade) and recommended restricting its concentration to less than 10 ng/mL in the natural water bodies (US Environmental Protection Agency 1976). *p*-Nitrophenol (PNP) is an important nitroaromatic compounds, and is used in the large-scale synthesis of acetaminophen, an aspirin substitute, and in the production of pesticides such as methylparathion and parathion (Spain and Gibson 1991). In the environment, these pesticides are also considered to be a major source of PNP detected in the environment since they can be converted to PNP under the action of hydrolase (Samuel et al. 2014). PNP is considered as a persistent, toxic contaminant (Kulkarni and Chaudhari 2006; EPA 2005). The toxicology and carcinogenicity of PNP have been studied (Ahmed et al. 2015, 2021).

**Fig. 1:**
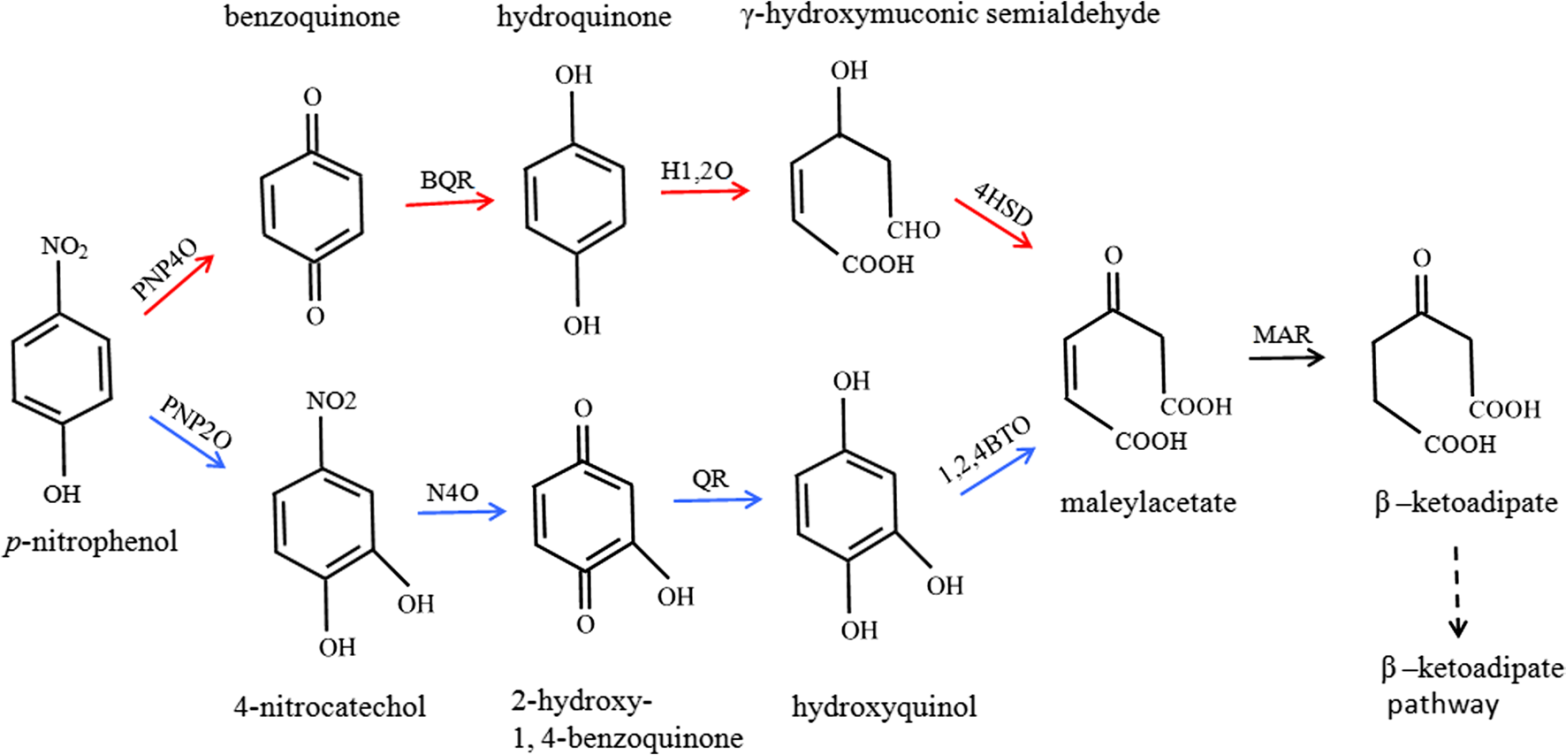
PNP degradation pathway and the two general pathways via hydroquinone or hydroxyquinol. PNP4O: *p*-nitrophenol 4-monooxygenase, (1.14.13.-); BQR: *p*-benzoquinone reductase, (1.6.5.-); H1,2O: hydroquinone 1,2-dioxygenase, (1.13.11.-); 4HSD: γ-hydroxymuconic semialdehyde dehydrogenase, (1.2.1.61); MAR: maleylacetate reductase, (1.3.1.32); N4O: 4-nitrocatechol 4-monooxygenase, (1.14.-); PNP2O: *p*-nitrophenol 2-monooxygenase, (1.14.13.29); QR: 2-hydroxy-1,4-benzoquinone reductase, (1.14.-); 1,2,4BTO: 1,2,4-benzenetriol dioxygenase, (1.13.11.37). The red arrows show enzyme actions designed for engineered strain, the dotted arrows show spontaneous reactions in bacteria

Different physical and chemical methods, including adsorption, electro or photo-catalyst have been used for removing these compounds (Vélez-Lee et al. 2016). But the disadvantages of these methods are sophisticated, expensive equipments and high energy consumption (Ma et al. 2014). Hence, biodegradation has been wildly concerned and become a hot topic due to its safety, low cost, minimal environmental impact and no secondary pollution (Peng et al. 2014; Zheng et al. 2009). Many microorganisms capable of degrading PNP have been isolated, such as bacteria (Zhang et al. 2008; Chauhan et al. 2010; Zhang et al. 2012; Spain 1995; Perry and Zylstra 2007; Shen et al. 2010) and microalgae (Lima et al. 2003), and their degradation pathways have been studied (Fig. 1). PNP would be converted to maleylacetate via hydroquinone pathway or hydroxyquinol pathway. In hydroquinone pathway, PNP was converted to maleylacetate via four enzymes (*p*-nitrophenol monooxygenase, benzoquinone reductase, hydroquinone 1,2-dioxygenase, dehydrogenase) which were encoded by *pnpA* to *pnpD*, respectively. In hydroxyquinol pathway, PNP was converted to 4-nitrocatechol via *p*-nitrophenol 2-monooxygenase, and then converted to 1,2,4-benezenetriol. Maleylacetate was converted to β-ketoadipate via maleylacetate reductase, which subsequently enters metabolites in a variety of anabolic pathways, including the TAC cycle and fatty acid biosynthesis (Wells and Ragauskas 2012).

For the first time, an *E. coli* strain was successfully constructed in this study to directionally degrade toxic PNP into carbon source by using the method of synthetic biology. The genes were selected from PNP-degradation bacterial *Pseudomonas putida*. Codons were modified and optimal designed according to the codon bias for engineered strain. Then, the PNP-degradation module containing the five genes involved in PNP degradation was constructed using T7 transcript system in a monocistronic transcriptional pattern. The module was then inserted into one vector after proving the bioconversion from PNP to β-ketoadipate for generating the recombinant PNP-degrading *E. coli* strain. In the remodeled *E. coli* cells, PNP was successfully converted to β-ketoadipate, and subsequently imported into the TCA cycle. The remodeled PNP-degraded strain can be used as a functional strain for the bioremediation of PNP and potential toxic intermediates. The method adopted in the study can also be used for transforming other strains or constructing engineered strains to create ones that can adapt to different environments or break down other pollutants.

## Materials and methods

### Reagents

*p*-Nitropheol was obtained from Aladdin (http://www.aladdin-e.com/zh_cn/). β-Ketoadipate was obtained from Finetech Industry Limited (Wuhan, China). All other chemicals were purchased from Sangon Biotech Co., Ltd. (Shanghai, China). Primers were synthesized from Sangon Biotech Co., Ltd. KOD DNA polymerase was purchased from TOYOBO Co., Ltd. (Osaka, Japan). Restriction enzymes were purchased from Takara Biomedical technology Co., Ltd. (Beijing, China).

### Bacteria and growth condition

The bacterium used for engineering construction was *E. coli* BL21-AI (Invitrogen, Waltham, MA, USA).

The basic liquid medium M9 (Na_2_HPO_4_·7H_2_O, 12.8 g/L; KH_2_PO_4_, 3 g/L; NH_4_Cl, 1 g/L; NaCl, 0.5 g/L) was used to culture *E. coli* BL21-AI, supplemented with glycerin (replace glucose) of 10 g/L, casamino acids of 1 g/L and thiamine hydrochloride of 10 ppm. In addition, the inducer isopropyl-β-d-thiogalactoside (IPTG) of 1 mM and arabinose of 2 g/L were also added to the medium for inducing gene expression.

*Escherichia coli* BL21-AI strain was incubated on a rotary shaker (160 rpm, 37 °C). The cells were harvested by centrifugation and weighed, washed three times with M9 and then re-suspended in the prepared inoculums (OD_600_ = 0.5) for subsequent use.

### Vector construction

The conversion of PNP to β-ketoadipate requires five genes, *pnpA* to *pnpE*. These genes from *Pseudomonas putida* (GenBank: FJ376608.2) were selected for chemical synthesis using PCR-based two-step DNA synthesis (PTDS) (Xiong et al. 2004). The codons of these five genes were modified and optimal designed to make them more suitable for *E. coli*. At the same time, stem loop structure, reverse repeat sequence and transcriptional terminator were eliminated to ensure mRNA stability and balance of GC content. The five synthetic genes, *pnpA*, *pnpB*, *pnpC*, *pnpD* and *pnpE* were renamed as *pnpAS*, *pnpBS*, *pnpCS*, *pnpDS* and *pnpES* (GenBank: MZ393850, MZ393851, MZ393852, MZ393853, MZ393854, respectively).

Each synthetic gene was connected between T7 promoter and terminator respectively, and arranged in the correct order. The gene expression cassette was then constructed using the polyacrylamide gel electrophoresis (PAGE)—mediated overlap extension PCR method (Peng et al. 2006). The constructed gene expression cassettes of the five gene was designated as T7*pnpAS*–T7*pnpBS*–T7*pnpCS*–T7*pnpDS*–T7*pnpES*, abbreviated as T7*pnpAS*–T7*pnpES*. Meanwhile, EcoRI and HindIII restriction sites were added to the 5′ and 3′ end of the expression cassette. The constructed expression cassette was then inserted into the expression vector pCAMBIA1301. Finally, the five-gene construction was completed and named as pYB3847.

### *Escherichia coli* transformation

The final pYB3847 plasmid was transformed into the host *E. coli* strain BL21-AI. The transformant was named as BL-PNP.

### Gene expression analysis

The transformant (BL-PNP) carrying the pYB3847 plasmid with all the five genes was used for RNA extraction. After 6 h of induction, total RNA from the transformant BL-PNP was extracted using TRIzol reagent (Invitrogen) according to the manufacturer’s instruction. Removal of genomic DNA and synthesis of cDNA was using cDNA Synthesis superMix (TransGen Biotech Co., Ltd., Beijing, China) according to the manufacturer’s instruction. The fluorescent quantitative PCR reaction (RT-PCR) of five genes was performed according to the method of Wang et al. (2019). The sequences of primers for each gene used are listed in Additional file 1: Table S1.

### *p*-Nitrophenol biodegradation

Different concentrations of *p*-nitrophenol (1 mM, 5 mM, 10 mM) were added to the prepared bacterial suspension containing inducers to detect biodegradation capability of the transformed strains. The strains transformed with empty vector under the same conditions were used as control group. M9 medium containing inducers but without any strains was used as blank group.

### Analysis of main metabolites of *p*-nitrophenol degradation

Cell densities were measured with optical densities at 600 nm (OD600) using an Infinite 2000 (TECAN) plate reader.

The concentration of PNP and its main hydrolysis, hydroquinone (HQ) and β-ketoadipate, were monitored over a 3-day period.

The concentration PNP and HQ were determined by High performance liquid chromatography (HPLC) using Agilent 1100 HPLC system (Agilent Technologies, CA, USA), which was equipped with Athena 5 μm C18 column (4.6 × 150 mm, CNW) (ANPEL Inc., Shanghai, China). The tested sample was twenty microliters. For PNP analysis, the mobile phase was methanol (50:50) at a flow rate of 0.5 mL/min. For hydroquinone analysis, the mobile phase was methanol (30:70) at 0.5 mL/min. PNP and HQ were detected at 318 nm, 270 nm respectively, using ultraviolet spectrophotometric detector (Agilent 1100 VWD).

The concentration of β-ketoadipate was detected by gas chromatography mass spectrometry (GC–MS) using the method of Okamura-Abe et al. (2016) and Wang et al. (2019) with minor modification. Samples were detected after derivatization. GC–MS analysis was performed on GC–MS/MS 7890B-7000C system (Agilent) equipped with a HP-5 MS column (30 m × 0.25 mm × 0.25 μm, Agilent). The oven parameters were: 40 °C/min from 100 to 160 °C, 10 °C/min from 160 to 250 °C, 20 °C/min from 250 to 300 °C.

### Statistical analysis

All experiments were repeated three replicates for each sample. Statistical significance was tested by Student’s t-test.

## Results

### Identification of PNP-biodegradation genes

PNP biodegradation pathway via the hydroquinone (HQ) pathway under aerobic conditions was chosen, the degradation pathways were shown in Fig. 1. Designing and optimizing the candidate genes to make them more suitable for *E. coli* was the first step of this experiment. Five genes, *pnpA* to *pnpE* for PNP degradation from *Pseudomonas putida* were selected for chemical synthesis, and these codons were designed and optimized according to preferential codon usage for *E. coli* in order to be conducive for improving gene expression (Additional file 1: Table S1). The identity of the synthetic genes showed 75.6%, 35.39%, 80.32%, 80.66% and 79.15% similarity with the original sequence.

### Vector construction and genetic transformation

**Fig. 2:**
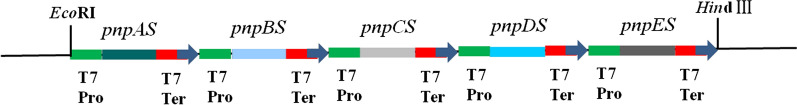
Schematic representation of recombinant vectors used in *E. coli* transformation. *pnpAS* (*p*-nitrophenol 4-monooxygenase), *pnpBS* (*p*-benzoquinone reductase), *pnpCS* (hydroquinone 1,2-dioxygenase), *pnpDS* (γ-hydroxymuconic semialdehyde dehydrogenase), *pnpES* (maleylacetate reductase)

The T7 promoter and terminator were selected for controlling the expression of each exogenous genes, as T7 phage RNA polymerase promoter was one of the strongest expression systems for expressing exogenous genes and recombinant proteins in *E. coli* (Landick 2004), The monocistronic transcriptional pattern was used for constructing the artificial gene cluster, the recombinant vector, which contained five lined genes in the committed PNP biodegradation pathway, was showed in Fig. 2.

### Expression of exogenous genes in the engineered strain

**Fig. 3:**
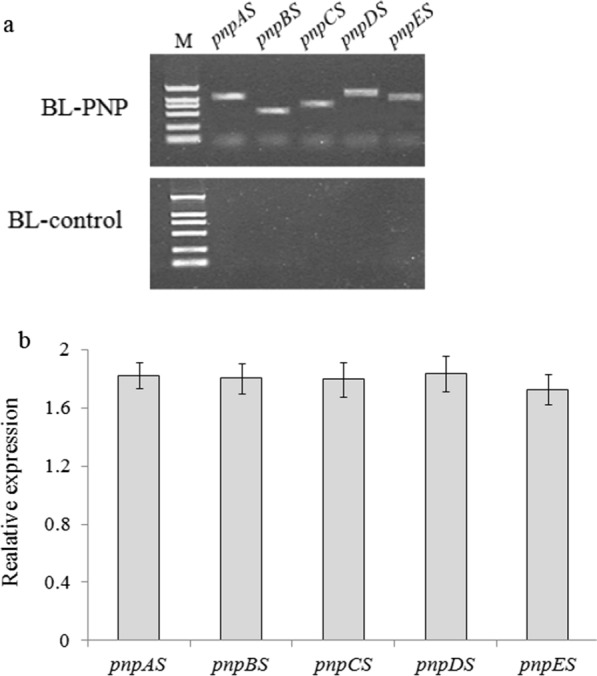
**a** Expression of transgenes by PCR using the plasmid extracted from BL-control or BL-PNP as template (M, DL2000). **b** Expression of transgenes by quantitative real-time PCR using cDNA of BL-control or BL-PNP as template

Genetically modification of multigene transformation is being accepted as an approach to generate microorganisms for environmental bioremediation. But the multigene vector will become more cumbersome and unstable as the increase of transgene (Zorrilla-López et al. 2013). The cDNA from the engineered strain were isolated and analyzed to verify whether the exogenous genes were stable. Results indicated that the expression vector with five genes was successful constructed and the expression of each gene was stable (Fig. 3a). The transcript expressions of the five genes were then analyzed through Real-Time PCR. Except the wild-type strain, all the five genes can be detected in the engineered strain BL-PNP (Fig. 3b). The RT-PCR demonstrated that the five genes were stably and actively transcribed in the engineered strain BL-PNP.

### Analysis of PNP degradation

**Fig. 4:**
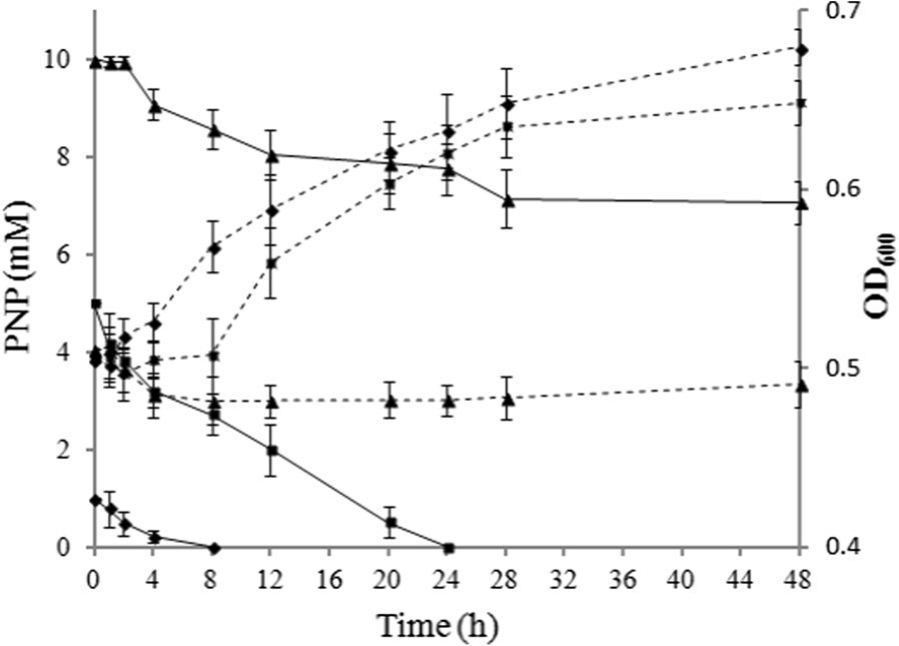
Degradation of PNP with different concentrations in the engineered strain BL-PNP, and the growth of the strain in the M9 media containing different concentration of PNP. The solid lines show the degradation of PNP, and the dotted lines show the growth of the engineered strain. Black diamond, 1 mM PNP; black square, 5 mM PNP; black up-pointing triangle, 10 mM PNP

The degradation ability of engineered strain BL-PNP at high concentrations of PNP was studied by treating them with different concentration of PNP. As shown in Fig. 4, PNP was completely degraded within 8 h and 24 h from an initial concentration of 1 mM and 5 mM, respectively. However, a lag period of 8 h was observed when the initial concentration was 5 mM. When the concentration reached 10 mM, only less than 3 mM PNP was degraded in 24 h, and the growth of BL-PNP was inhibited.

### Metabolites analysis

In this study, the engineered strain was designed to completely degrade PNP via the pathway of hydroquinone (HQ), thus the metabolites HQ and the last hydrolysis product, β-ketoadipate, were analyzed.

**Fig. 5:**
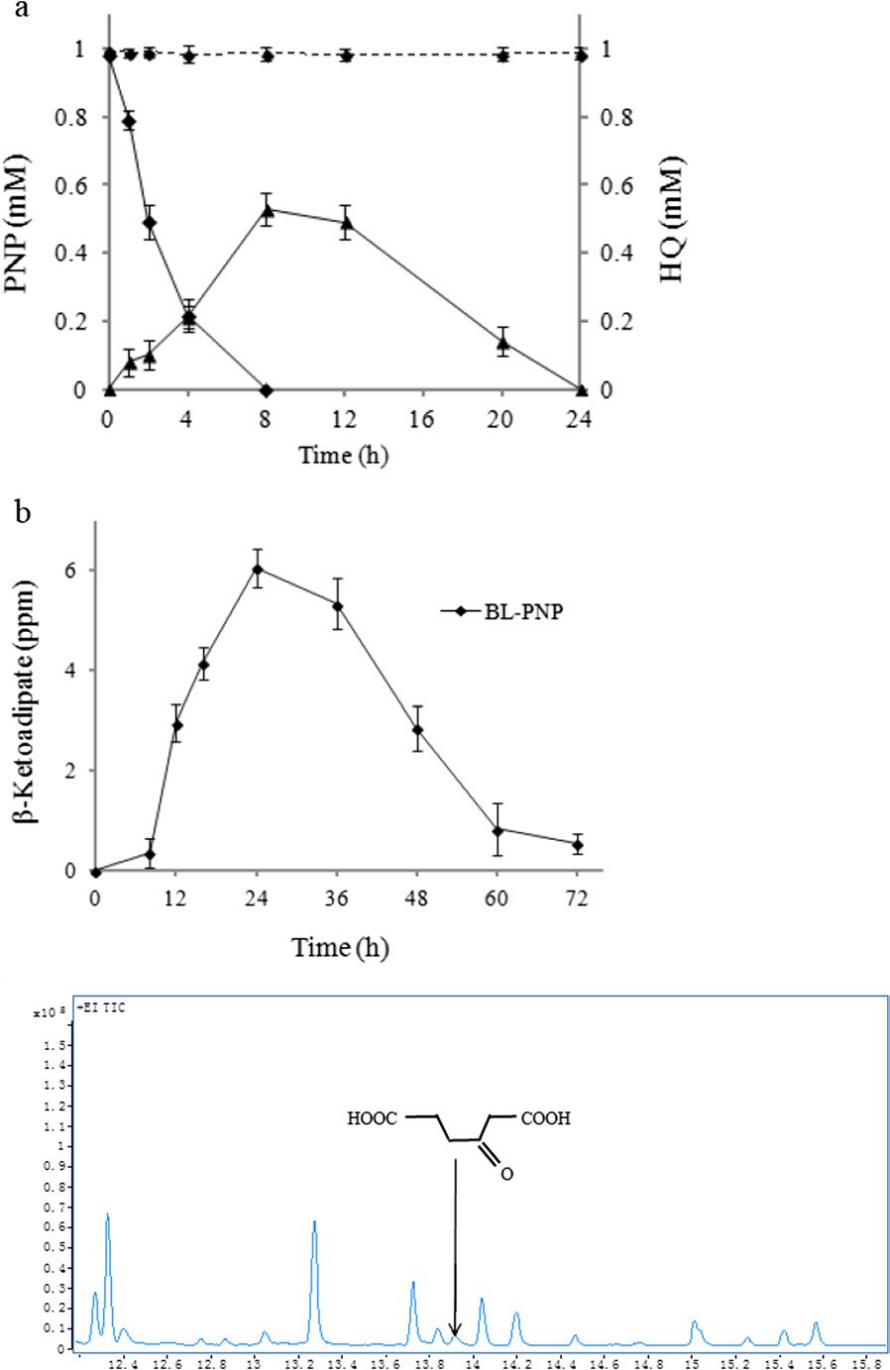
Degradation of 1 mM PNP. PNP was converted to β-ketoadipate via HQ pathway as designed. **a** Degradation of PNP and formation of HQ. The solid lines show the change of the metabolites in the engineered strain BL-PNP, and the dotted lines show the one in control strain. Black diamond, the degradation of PNP; black up-pointing triangle, the formation of HQ. **b** GC–MS analysis of β-ketoadipate concentration at different times in the engineered strain BL-PNP

When the concentration of PNP was 1 mM, it can be rapidly degraded in 8 h. At the same time, with the depletion of PNP, the concentration of HQ was increased gradually and peaked at 8 h (Fig. 5a). However, the amount of PNP hydrolysis and HQ generation were not stochiometric. After the peak, the concentration of HQ gradually decreased and 74% of HQ was degraded within 24 h. It cannot be detected after 24 h in the degradation system.

β-Ketoadipate, the last degraded product constructed in the experiment, was also detected. When 1 mM PNP was added to the liquid medium, the concentration of β-ketoadipate was increased slowly within the first 8 h. It then increased rapidly within 24 h, and peaked at 24 h. After that, the concentration of β-ketoadipate gradually decreased (Fig. 5b). The results illustrated that the pathway for the complete degradation of PNP in *E. coli* has been successfully constructed, and the β-ketoadipate produced by the degradation of PNP can quickly enter into multiple anabolic pathways. Therefore, the engineered strain BL-PNP will be useful for the bioremediation of the phenolic compounds.

In BL-control strain, the content of PNP was detected with the same period of time as BL-MP within 3d, and no degradation was found (Fig. 5a). The PNP hydrolysis products, hydroquinone and β-ketoadipate, were not found, neither (data not shown).

## Discussion

As a commercial commodity, nitrophenols are widely used in the production of herbicides, pesticides and other aspects (Kulkarni and Chaudhari 2007), which led to serious environmental pollution. Microorganisms, especially bacteria living in various environments can grow in the environment containing nitrophenol compounds due to their unique ability, and at the same time, they also achieve the purpose of degradation of nitrophenol. In addition, genetically modification of bacterial strains is one of the useful tools to improve the degradation of nitroaromatic compounds (Nordin et al. 2005; Xiao et al. 2007). Thus, searching novel strains or constructing modified ones which are capable of removing environmental contaminant is a very interesting academic task (Frey and Kallio 2003). In this study, a modified strain which can completely biodegrade PNP was first constructed with modulated PNP degradation pathways by using the method of synthetic biology.

Traditionally, the way to remove toxic contaminants was to use microorganisms found in nature, but the disadvantage was that the background is too complex to effectively improve the degradation efficiency. *E. coli*, due to its clear background, favorable growth condition, and availability of versatile genetic manipulation tools, has become an ideal platform host for biosynthesis and biodegradation (Pósfai et al. 2006; Pontrelli et al. 2018). Biodegradation has also become easier and more suitable for engineering by establishing a series of standard biological operating units, such as promoters, operons, resistance screening markers, and some regulatory factors, etc. (McNerney et al. 2015; Anderson et al. 2010).

Metabolic engineering researches in prokaryotes are mainly based on operon modification (Chhabra and Keasling 2010). The simultaneously coordinated expression of multiple genes is the most essential requirement for the construction of multigene engineering in heterologous microbial background. In transgenic plants, multiple transgenes controlled by the same promoter have shown coordinated and stable expression when the same promoter used repeatedly for each gene (Zhu et al. 2008; Tian et al. 2020). In transgenic microorganism, the same operation can also be used to achieve the same goal, which has been demonstrated in our laboratory, where we have successfully constructed a bacterial multigene engineered strain and achieved the biodegradation of phenol (Wang et al. 2019).

Due to the cytotoxicity of PNP, most of the studies mainly focused on the degradation of PNP at lower concentrations (10 ng–150 ppm). In this study, the degradation of PNP at high concentrations was studied, and the engineered strain BL-PNP had high tolerance and rapid degradation ability among the reported PNP-degrading strain. Phenolic compounds have been reported to have exerted toxic effects on membrane owing to their high aqueous solubility (Sikkema et al. 1995), which leads to a lag phase in the degradation of high concentration of PNP. *P. putida* has been reported to completely degrade 300 ppm and 500 ppm PNP within 36 h and 72 h with a lag period of 12 h and 20 h, respectively (Kulkarni and Chaudhari 2006; Samuel et al. 2014). Similar reports have been reported for other bacteria, such as *Stenotrophomonas* (Liu et al. 2007), *Pseudomonas aeruginosa* (Zheng et al. 2009). This could be due to the antimicrobial toxicity and dose dependent of PNP (Bhushan et al. 2000; Orenes-Piñero et al. 2013). The engineered strain BL-PNP had a shorter lag phase and faster degradation rate, indicating that BL-PNP can quickly adapt to the toxic concentrations of organic substrate. These characteristics made it potentially useful for the PNP-biodegradation of high concentrations.

As designed, PNP was completely degraded via the pathway of hydroquinone (HQ), which was the common metabolic of hydroxylation of *p*-substituted phenols (Spain and Gibson 1991; Bae et al. 1996). However, the amount of PNP degradation and HQ generation were not stochiometric. This might be due to the immediate metabolism of HQ, which was constantly produced by the hydrolysis of PNP and continuously degraded at the same time. β-Ketoadipate was designed as the last degraded product constructed in the experiment. The β-ketoadipate pathway, which is an enzyme-mediated aryl-ring degradation sequence, was widely employed by soil bacteria and fungi to convert many harmful aromatic pollutants into benign ones, including TCA (tricarboxylic acid cycle) metabolites, lipogenesis and other anabolic processes (Ju and Parales 2010). The decrease of β-ketoadipate produced by the degradation of PNP illustrated that it can quickly enter into multiple anabolic pathways and the engineered strain BL-PNP will be useful for the bioremediation of the phenolic compounds.

Here, *E. coli* obtained a new PNP-biodegradation function by precisely designing metabolic pathways. However, the degradation efficiency of toxic pollutants by engineered strains in the field condition could mostly insufficient compared with laboratory conditions. The main reasons are as follow: availability of oxygen and competition with autochthonous microorganisms. Therefore, it is necessary to consider some factors for biodegradation, such as location, cost, environmental types and policies, and so on (Azubuike et al. 2016). In addition, the ecological risks associated with bioremediation using engineered strains, such as gene flow, competition and fitness should also be evaluated and controlled (Kuiken et al. 2014).

## Supplementary Information

**Additional file 1: Table S1.** The sequences of primers for respective gene used in this study.

## Data Availability

The datasets generated during and/or analysed during the current study are available from the corresponding author on reasonable request.
