# Supplementary material for: Biodegradation of p-nitrophenol by engineered strain
Source: AMB Express. 2021 Aug 31;11:124. doi: 10.1186/s13568-021-01284-8 (PMC8408293; doi:10.1186/s13568-021-01284-8)
Supplement: Supplementary file 1 — Additional file 1: Table S1. The sequences of primers for respective gene used in this study. [file 13568_2021_1284_MOESM1_ESM.docx]

AMB Express

Biodegradation of *p*-nitrophenol by engineering strain

Jing Xu, Bo Wang, Wen-hui Zhang, Fu-Jian Zhang, Yong-dong Deng, Yu Wang, Jian-Jie Gao, Yong-Sheng Tian*, Ri-He Peng*, Quan-HongYao*

a Shanghai Key Laboratory of Agricultural Genetics and Breeding, Agro-Biotechnology Research Institute, Shanghai Academy of Agricultural Sciences, Shanghai 201106, China

* Corresponding author

E-mail addresses: Yong-Sheng Tian: tys810508@126.com,

Ri-He Peng: pengrihe@163.com,

Quan-HongYao: [yaoquanhong_sh@aliyun.com](mailto:yaoquanhong_sh@aliyun.com).

Jing Xu and Bo Wang are contributed equally to the article.

Additional File 1

Table S1: The sequences of primers for respective gene used in this study

| Name | Forward Primer | Reverse Primer |
| --- | --- | --- |
| *pnpAS* | 5´-CAACTGTGCGTGGTAAGATC-3´ | 5´-CGACCACGACGGAACTCAG-3´ |
| *pnpBS* | 5´-CAGATGCGTAACTTCCTTG-3´ | 5´-CAGGACCAGCAAGAGTTG-3´ |
| *pnpCS* | 5´-TGGTCGTTACCGTTCTGAC-3´ | 5´-GTCTTCGTACTTCACTTGC-3´ |
| *pnpDS* | 5´-TGGGTCCACTGACTTCTG-3´ | 5´-GTCCACAGACCAGAACCCA-3´ |
| *pnpES* | 5´-CGTGGATGACCCTGAACA-3´ | 5´-GGGATACCAAGTTTCTCG-3´ |
